# Current understandings in treating children with steroid-resistant nephrotic syndrome

**DOI:** 10.1007/s00467-020-04476-9

**Published:** 2020-02-21

**Authors:** Jiwon M. Lee, Andreas Kronbichler, Jae Il Shin, Jun Oh

**Affiliations:** 1 Department of Pediatrics, Chungnam National University Hospital, Daejeon, South Korea; 2 Department of Internal Medicine IV (Nephrology and Hypertension), Medical University Innsbruck, Innsbruck, Austria; 3 Department of Pediatrics, Yonsei University College of Medicine, 50 Yonsei-ro, Seodaemun-gu, C.P.O. Box 8044, Seoul, 120-752 South Korea; 4 Division of Pediatric Nephrology, Severance Children’s Hospital, Seoul, South Korea; 5 Institute of Kidney Disease Research, Yonsei University College of Medicine, Seoul, South Korea; 6 Department of Pediatrics Nephrology, University Hamburg-Eppendorf, Martinistrasse, 52 20246 Hamburg, Germany

**Keywords:** Nephrotic syndrome, Resistant, Intractable, Monogenic, Treatment, Children

## Abstract

Steroid-resistant nephrotic syndrome (SRNS) remains a challenge for paediatric nephrologists. SRNS is viewed as a heterogeneous disease entity including immune-based and monogenic aetiologies. Because SRNS is rare, treatment strategies are individualized and vary among centres of expertise. Calcineurin inhibitors (CNI) have been effectively used to induce remission in patients with immune-based SRNS; however, there is still no consensus on treating children who become either CNI-dependent or CNI-resistant. Rituximab is a steroid-sparing agent for patients with steroid-sensitive nephrotic syndrome, but its efficacy in SRNS is controversial. Recently, several novel monoclonal antibodies are emerging as treatment option, but their efficacy remains to be seen. Non-immune therapies, such as angiotensin-converting enzyme inhibitors or angiotensin II receptor blockers, have been proven efficacious in children with SRNS and are recommended as adjuvant agents. This review summarizes and discusses our current understandings in treating children with idiopathic SRNS.

## Introduction

Nephrotic syndrome is the most common glomerular disease in childhood. The prevalence is 12–16 per 100,000 children aged under 16 [1]. The reported annual incidence in children varies between 1.2 and 3.5 per 100,000 per year in Western Europe [2–4], 4.7 per 100,000 per year worldwide [5] and up to 6.5 per 100,000 per year in Japan [6]. Although more than 85% of children with nephrotic syndrome respond to corticosteroids, approximately 10–15% remain unresponsive or later become steroid-resistant [7–11]. Steroid-resistant nephrotic syndrome (SRNS) has been associated with unfavourable renal prognosis, with 36–50% of patients progressing to end-stage kidney disease within 10 years [12–14].

Standardized management of SRNS has been hampered by the paucity of high-quality trial evidence. SRNS is rare, and as such treatment options are based on anecdotes, case reports mainly illustrating individual centre practice and studies with relatively small numbers of patients. Protocols are therefore generally individualized using a broad framework. In addition, there are few clinical studies with a sufficient power in children with SRNS.

## Recent perspectives on the stratification of nephrotic syndrome

For many years, idiopathic nephrotic syndrome has been classified based on the patient’s response to steroids: as steroid-sensitive or steroid-resistant. SRNS, most commonly focal segmental glomerulosclerosis (FSGS) in histology, has long been thought as an immune-mediated disease, either with or without circulating factors. However, recent techniques have enabled the discovery of a multitude of genetic mutations that cause nephrotic syndrome. Because this genetic disease group is not immune-mediated, it is theoretically unresponsive to steroids or other immunosuppressants and thus is classified as SRNS. Monogenic, non-immune-mediated causes reportedly account for up to one third of SRNS cases overall, and to date more than 60 genes related to SRNS have been described [15, 16]. Therefore, SRNS is now a rather heterogeneous constellation of distinct aetiologies. As to this, a recent comprehensive review by Saleem [15] has proposed a novel molecular stratification of SRNS based on disease mechanisms as monogenic, immune-mediated with circulating factors and immune-mediated without circulating factors.

This review attempts to appreciate the newly proposed stratification by summarizing the treatment of monogenic SRNS in a separate section; however such efforts are limited because most publications have not (or could not at the time) stratified patients by their genetic bases. Nevertheless, this review attempts to summarize current understandings and controversies in therapies available for children with idiopathic (primary) SRNS.

## Intensified immunosuppression and adjuvant agents for SRNS

### Intravenous methylprednisolone pulse

The mechanism of action of steroid therapy in inducing remission is complex. Its main actions are immune modulation by binding to specific cytoplasmic receptors, modifying transcription and protein synthesis and thereby suppressing inflammatory reactions and humoral immune response [17, 18]. The rationale for administration of intravenous methylprednisolone pulse despite initial steroid unresponsiveness is based on the results from observational studies which reported remission in steroid- and/or cyclosporine-resistant nephrotic syndrome. Previous studies reported remission induced in SRNS using methylprednisolone pulses with concomitant immunosuppressants [19–21] and by methylprednisolone alone [22, 23]. Specifically, Mori et al. reported remission after methylprednisolone pulse therapy in 78% (7/9) of patients who were resistant to steroids and cyclophosphamide (CPM) and/or cyclosporine A (CSA) [23]. The studies showed that prolonged methylprednisolone pulses with alternate-day oral steroids may be efficacious, even when patients are considered to have steroid- and multidrug-resistant nephrotic syndrome. Mechanisms leading to such a late remission following steroid pulses despite initial resistance need to be elucidated [12].

However, several studies argue against the use of prolonged high-dose steroids in SRNS [9, 24, 25], given the adverse effects of long-term steroid use. In addition, in the PodoNet registry, a large web-based clinical database in Europe for idiopathic SRNS including congenital nephrotic syndrome and high-dose steroids (including pulses) used as first-line therapy were not efficacious in ~ 85% [9]. Still, if there was a partial remission seen with conventional oral prednisolone therapy, it appears to be worth trying intravenous methylprednisolone pulses as combination therapy.

### Calcineurin inhibitors

There are two mechanisms of the antiproteinuric effect exerted by calcineurin inhibitors (CNI) discussed (1) through inhibition of T-cell signalling in lymphocytes and (2) direct non-immune effects on the podocyte actin cytoskeleton [7, 26]. The latter part of the mechanism explains the partial efficacy of CNIs in non-immune-mediated, monogenic SRNS, reported in the literature [27]. Complete or partial remission rates in SRNS with the use of CNIs have been in the range of 30–80% in observational studies [11] and randomized controlled trials (RCTs) [28, 29].

A multicentre RCT [29] showed that CNIs significantly increased the proportion of children with SRNS obtaining remission, compared with intravenous CPM. This finding was supported by a systematic review of RCTs by Hodson et al., which showed that CSA was effective irrespective of renal pathology compared with placebo and intravenous CPM [30].

Combination of steroids and CNIs has been proven efficacious for treating SRNS in systematic reviews [28, 30] and is currently recommended by the Kidney Disease Improving Global Outcomes (KDIGO) guideline [12]. The guideline recommends continuation of CNI in SRNS for a minimum of 12 months [31]. This statement was supported by a study highlighting the benefits of continued treatment with tacrolimus (TAC) for 18 months [32]. There is, however, no international consensus on duration of treatment or optimal target trough levels.

While other CNIs like voclosporin are currently being tested in clinical trials, CSA and TAC are the two major agents which are commonly used in the treatment of nephrotic syndrome. TAC and CSA are generally accepted to have similar therapeutic effects [7], showing similarly superior efficacy over CPM [29] and no significant difference in the numbers of SRNS patients achieving remission [30, 33]. Prasad et al. studied 45 children with SRNS who were resistant to CPM, in which they treated the patients with either CSA or TAC and compared treatment outcome at 6 months [33]. They showed that CNIs were effective in CPM-resistant nephrotic syndrome and reported comparable results of TAC and CSA in inducing remission in 70% and 82% of patients, respectively (*P* = 0.3) [33]. However, Choudhry et al. showed that significantly fewer children relapsed in the TAC-treated group compared to the CSA-treated group [34], suggesting superior efficacy of TAC over CSA.

Concerns exist regarding chronic nephrotoxicity with the use of long-term CNI. Continuation of CNIs for more than 2 to 5 years has been shown to be associated with renal toxicity [32, 35, 36]. In patients with steroid-dependent nephrotic syndrome (SDNS), chronic histologic lesions due to CNI use were reported in as high as 35–75% of patients on renal biopsy [37–40]. For SRNS, Fujinaga et al. performed protocol biopsies in six SRNS children who used CSA for more than 2 years, and 87% (5/6) had nephrotoxic histologic changes [41]. These figures, however, are not directly comparable because the time points to perform a renal biopsy are not uniform between the studies. Still, it appears that incidence of nephrotoxicity is higher in patients with SRNS compared to those with SDNS [42].

With regard to nephrotoxicity, TAC appears to have a lower potential risk [43], and a multicentre study on SRNS patients has suggested a better preserved, long-term renal function in the TAC-treated group compared to CSA treatment [13], showing less arteriolopathy and interstitial fibrosis on renal biopsy [14]. In the study by Prasad et al. [33], 45 children with SRNS treated with either CSA or TAC were compared for renal survival at 1, 2, 3, 4 and 5 years. The results demonstrated that TAC had significantly superior outcomes in terms of long-term renal function (*P* = 0.02) [33]. Moreover, a study by Delbet et al. in 21 paediatric SDNS patients on CNI for more than 12 months found relatively infrequent (only 1 out of 21) renal histology of CNI nephrotoxicity, which the authors explained by a greater use of TAC over CSA [43]. Furthermore, in an RCT with 124 renal transplant recipients, conversion from CSA to TAC resulted in stabilized serum creatinine levels (whereas the CSA-continuation group had a significantly greater rise in serum creatinine) and resulted in a sustained reduction of blood pressure, improvement in serum lipid profile and quality of life [44]. In contrast, another study on renal transplant recipients reported comparable incidence of arteriolopathy between TAC and CSA [45].

Nevertheless, most centres have changed their prescription pattern towards TAC, as it appears to exhibit better efficacy and less systemic side effects, particularly nephrotoxicity. In addition, compared to CSA, TAC has fewer cosmetic side effects, such as gingival hypertrophy and hirsutism [34]. RCTs comparing both agents with a long-term follow-up are necessary to specifically address these points, and generalizable recommendations may be extrapolated thereafter.

### Cyclophosphamide

CPM is an alkylating agent lowering the immune response and thereby used in several autoimmune diseases. It has been proven effective in maintaining remission and reducing relapses in steroid-sensitive nephrotic syndrome (SSNS). For SRNS, however, a body of literature suggested its limited efficacy in inducing sustained remission [8, 9, 46–50], and there are currently no RCTs advocating the use of CPM in SRNS. Instead, CNIs are accepted for superior efficacy to CPM in the management of SRNS. A comparative study on the treatment effects of CPM and CSA was performed in 127 children with SRNS. At 5 years of follow-up, a higher response rate has been observed in 65 children receiving CSA for 2 years compared to 62 patients assigned to CPM treatment for 3 to 6 months (70.8% vs. 51.6%, *P* = 0.027), and a relapse-free survival also favoured CSA over CPM (38.3 vs. 32.5 months, *p* < 0.001) [51]. Similarly, superior effects were seen with TAC, showing significantly shorter mean time to remission compared with CPM [29]. These findings were supported by a systematic review showing that CNIs significantly increased the number of patients achieving remission compared with CPM [30]. In this systematic review, CPM plus steroids had no benefit compared to steroid monotherapy [30]. Moreover, with its substantial side effect, spectrum needs to be considered, such as gonadal dysfunction, myelosuppression and increased long-term risk of malignancy [52]. Thus, the use of CPM for SRNS is rather discouraged and officially not recommended by the KDIGO guidelines (Table 1) [12].

**Table 1:** Efficacy of treatment options used in the management of cases with SRNS

Drug	Dose (may differ by centres)	Efficacy	Recommendation^a^
RAS inhibitors	–	In 40–50% lowered PU [53, 54] CR in 27%, PR in 21% [9]	Yes [7, 8, 53, 54]
Steroid pulse	30 mg/kg (max.1 g) QOD Mendoza protocol for 82 weeks	No effect in ~ 85% [9] CR or PR in 33–53% [12, 48, 55] CR in 7%, PR in 11%, no effect in 83% [11]	No [9]
Cyclophosphamide	IV 500 mg/m^2^ PO 2 mg/kg/d (max. cumulative dose 168 mg/kg)	No effect [12] CR in 9%, PR in 8%, no effect in 83% [11]	No [8, 12]
Mycophenolate mofetil	PO 1200 mg/m^2^/d #2	CR 23–62%, PR 25–37% [12, 52, 56] CR in 8%, PR in 8%, no effect in 83% [11]	No for monotherapy [11, 30, 55] Yes for triple therapy [57]
Calcineurin inhibitors	Cyclosporine 4–6 mg/kg/day #2 (trough 150–300 mg/ml) Tacrolimus 0.1–0.2 mg/kg/day #2 (trough 5–15 μg/ml)	CR or PR in 70% [7] CR or PR in 50–70% [7] CR or PR in 30–80% [28] CR in 30%, PR in 19% [11]	Yes [7, 28, 30]
Rituximab	IV 375 mg/m^2^ weekly or biweekly	CR in 44%, PR in 15% [9] CR or PR in 46% [58] No benefit [59]	Yes [60–62] No [59, 63]
Ofatumumab	Variable 300 mg/1.73m^2^ → 2000 mg/1.73m^2^ [64] 750 mg/1.73m^2^ [65] 300 mg/1.73m^2^ [66]	CR or PR in 5/5 pt. [67] CR or PR in 2/4 pt. [68] CR or PR in 4/4 pt. [64]	Little evidence
Abatacept/Belatacept	Abatacept 10 mg/kg once or twice daily	CR or PR in 5 pt. [69] No effect in 1 pt. [70]	Little evidence
Adalimumab (mAb TNF-α)	SC 24 mg/m^2^ (max.40 mg) biweekly	Phase I trial: PR in 40% [71] Phase II trial: no response [72]	No (Phase II) [53]
Fresolimumab (mAb TGF-ß)	1 mg/kg or 4 mg/kg	Phase I trial: CR or PR in 18% [53] Phase II trial: no response [73]	No (Phase II) [73]
Galactose	PO 0.2 g/kg (max.15 g) twice daily	Phase II trial: CR or PR in 40% [72] PR in 29% [72, 74, 75] No effect in PU [76]	Yes (Phase II) [72]
ACTH analogues	Gel 80 U/1.73 m^2^ twice weekly IM 1 mg weekly × 12 months	PR in 29% [77] CR in 33% (6/18) and PR in 22% (4/18) [78]	Little evidence
Sparsentan	200, 400, 800 mg/d	More efficacious than irbesartan after 8 weeks [79]	Little evidence

*CR* complete remission; *PR* partial remission; *mAb* monoclonal antibody; *IV* intravenous; *PO* per oral; *SC* subcutaneous; *QOD* every other day

^a^ Recommended by one of the followings: systematic reviews, meta-analysis, official guidelines, reviews, phase I or II clinical trials

### Mycophenolate mofetil

Mycophenolate mofetil (MMF) modulates the immune response by inhibiting inosine monophosphate dehydrogenase, a key enzyme involved in purine biosynthesis [26], and thereby inducing selective inhibition of DNA replication in T and B lymphocytes [7].

MMF has been effectively used as a remission-maintaining and steroid-sparing agent for children with SSNS, including frequently-relapsing nephrotic syndrome (FRNS) or SDNS [8, 80]. For SRNS, several observational studies using MMF have demonstrated complete remission in 23–62%, partial remission in 25–37% and no response in 8–40% of patients, although the results were largely impacted by a high likelihood of publication bias due to small numbers of patients [12, 56, 81, 82]. MMF has also been suggested to be effective in children with SRNS under the age of 2 years [56].

Contrastingly, in a large longitudinal study from the PodoNet registry, MMF monotherapy during the first year of disease onset in 612 SRNS patients led to no remission in most (83.3%) cases [11]. However, it should be noted that the registry contains both immune-mediated and monogenic SRNS. In addition, an RCT including children and adults compared dexamethasone (high cumulative exposure) plus MMF (DEX/MMF) to CSA monotherapy [55], showing no benefit of the former over the latter. In this study, sustained response was reported in 33.3% of subjects receiving DEX/MMF and 45.9% in those receiving CSA [55]. A systematic review also found no statistically significant difference in the number of remissions between a combination of MMF/steroids to CSA or TAC monotherapy [30]. Moreover, an open-label RCT demonstrated that MMF was inferior to TAC in sustaining remission in 60 children with SRNS [32]. In this study, in patients who had previously achieved complete or partial remission using TAC, a switch from TAC to MMF at 6 months failed to maintain remission, yielding a twofold increased incidence of relapses and leading to higher prednisolone exposure [32].

Still, MMF is sometimes preferred for its relative safety with respect to nephrotoxicity. In the National Institutes of Health (NIH)-funded FSGS study, the authors reported a significantly lower median glomerular filtration rate (GFR) at 6 months after treatment in FSGS patients receiving CSA compared with those receiving DEX/MMF [55], which was further supported by an RCT in SSNS [83]. However, an RCT in children with SRNS showed no difference in GFR between MMF- and TAC-treated patients [32].

In general, the efficacy of MMF in patients with SRNS appears less satisfactory than in those with SSNS [32] and seems not superior to CNI monotherapy in SRNS. Nevertheless, MMF can be effective as an additive agent in maintaining CNI-induced [84–86] and rituximab (RTX)-induced remission [87] in SRNS patients, although premature switching to MMF is not recommended [32]. MMF may also be an alternative in patients who are resistant to CNI, which will be discussed below.

### Levamisole

Levamisole is an immune-modulating anthelminthic that has been considered the least toxic and least expensive steroid-sparing agent for preventing relapses in SSNS [88]. Levamisole is a synthetic imidazole derivative which, instead of suppressing immunity, enhances humoral immune response and macrophage activation [89] and induces type 1 (Th1) and type 2 (Th2) T-cell responses through enhancing IL-18 activity [90]. In a human podocyte model, levamisole was shown to induce expression of glucocorticoid receptor, and it has been suggested that glucocorticoid signalling is a critical target of levamisole action [91].

Levamisole was shown to be effective in reducing the frequency of relapses in adult patients with SSNS [91], which was supported by a systematic review of RCTs [92]. In children with SSNS, a recent open-label RCT showed that levamisole had comparable and satisfactory efficacy compared to MMF in maintaining sustained remission [93].

With respect to SRNS, however, there is a paucity of data supporting the use of levamisole. In their experience with levamisole treatment in children with SSNS and SRNS, Tenbrock et al. concluded that levamisole had a clinical benefit in SSNS but not in SRNS [94]. Few studies thereafter have been performed in SRNS. In short, the efficacy of levamisole in SRNS so far appears limited.

### mTOR inhibitors

Mammalian target of rapamycin (mTOR) signalling is involved in a variety of kidney diseases. mTOR inhibitors are assumed to work through control of autophagy [95]. They block T-cell proliferation and bind to the same immunomodulators as TAC, without affecting the activity of calcineurin [96]. Low-dose rapamycin, an inhibitor of the mTOR, has been reported to diminish disease progression in an experimental model of FSGS [97]. Rapamycin has been successfully used in a case series including three patients with FSGS [98] and in ten children with SRNS [96]. Moreover, in a prospective, open-label trial of sirolimus to treat steroid-resistant FSGS, complete or partial remission was reported in 47% of the treated patients [99]. However, a phase II open-label clinical trial study on sirolimus had to be prematurely discontinued due to renal functional deterioration after administration [100]. In addition, mTOR inhibitors at high dose may rather cause aggravation of proteinuria in FSGS [100]. Given their potential detrimental renal effects, the use of mTOR inhibitors in cases of SRNS is discouraged.

### Therapy for CNI-dependent or CNI-resistant SRNS

A subset of SRNS patients is CNI-dependent, who respond to CNI treatment but relapse once therapy is tapered or discontinued, and another proportion is resistant to CNI. Since prolonged use of high-dose CNI can result in undesirable side effects (i.e. nephrotoxicity), alternative or additive drugs have been sought.

El-Reshaid et al. treated 21 CNI-resistant SRNS patients with CNI in combination with MMF and monthly intravenous CPM for a total of 3 pulses and induced complete remission in 71% (15/21) [101]. Former triple therapy approaches comprised CNI plus prednisolone and a third agent, such as MMF [102, 103] or mizoribine [104] (Table 2). Of these, MMF may be an effective third agent, supported by additional reports which show that remission was achieved in 20 to 50% of patients refractory to treatment with CNIs and corticosteroids [32, 105, 106]. Some retrospective case series have also showed that MMF, as a maintenance agent, enabled CNI- and steroid-free remission in some patients with CNI dependence and/or toxicity [32, 107, 108]. Still, as discussed in the previous section of this review, MMF as a monotherapy or in dual combination with prednisolone was not superior to CNIs in terms of inducing remission in SRNS [30, 32]. Wu et al. then performed a prospective RCT in 18 children with steroid- and TAC-resistant or TAC-dependent nephrotic syndrome [57]. They reported that triple-combination therapy with prednisolone, TAC and one out of MMF, CPM or leflunomide was effective for short-term response and remission at 1 year, with comparable efficacy between the three agents and without significant side effects [57]. However, prolonged intensive immunosuppression with a combined regimen may predispose patients to serious infectious complications and potential malignancy risk in the long term, and this should be closely monitored. Therefore, novel agents that could be used for a shorter period of time as pulse therapy have been sought, namely, monoclonal antibodies.

**Table 2:** Combination treatment for CNI-resistant or CNI-dependent SRNS

Ref	Patients	Agents	Response	F-U
Wu, 2015 [105]	10 TAC-resistant children 8 TAC-dependent children	Pd + TAC + MMF/CPM/leflunomide (3rd agents had equal efficacy)	Short term response 14/18 (78%) Long-term remission 11/18 (61%)	5 yr
El-Reshaid, 2005 [97]	21 CNI-resistant MCD/FSGS	CNI + MMF + IV CPM (for 3 months)	Complete remission 15/21 (71%)	6.5 yr
Ballarin, 2007 [98]	9 adults with MGN	Pd + TAC + MMF	Complete remission 2/9 Partial remission 3/9	23.1 mo
Oki, 2009 [100]	2 CSA-resistant children	Pd + TAC + mizoribine	2/2 complete remission	8 mo and 17 mo
Kim, 2014 [99]	8 refractory SRNS	Pd + TAC + MMF	75% response	N/A

*CR* complete remission; *PR* partial remission; *TAC* tacrolimus; *Pd* prednisolone, *MMF* mycophenolate mofetil; *CPM* cyclophosphamide; *F-U* follow-up; *CNI* calcineurin inhibitor; *MGN* membranous glomerulonephropathy; *MCD* minimal change disease; *FSGS* focal segmental glomerulosclerosis; *yr* years; *mo* months; *N/A* not available

## Anti-CD20 monoclonal antibodies

### Rituximab and novel CD20-blocking agents

Multidrug-resistant patients who are unresponsive to above listed agents – steroids, CNI and MMF – pose a great challenge for nephrologists. Monoclonal antibodies have been investigated as salvage therapy for these patients.

Rituximab (RTX) is a chimeric anti-CD20 monoclonal antibody which has been used as an alternative steroid-sparing agent for SSNS patients since the early 2000s and is generally well tolerated. Its main action on immune regulation is through targeting the cell surface antigen CD20 on B lymphocytes and inducing B-cell depletion [26]. In addition, a non-immune-related mechanism of action has been proposed. RTX reportedly could affect podocyte function through stabilization of sphingomyelin phosphodiesterase acid-like 3b (SMPDL-3b), preventing podocyte actin remodelling [109].

While RTX has been successfully used in patients with FRNS or SDNS in recent years [110], a comparative study by Topaloglu et al. explored the effects of RTX in children with SSNS (*n* = 21) and SRNS (*n* = 20) and concluded that RTX had more positive therapeutic effects in patients with SSNS than in those with SRNS [111].

Although controversial results exist related to its efficacy in SRNS, it has been generally successful in children with SRNS [60, 112]. Reportedly, approximately 50% of patients with refractory SRNS respond to RTX [61], showing complete remission in 44% and partial remission in 15% [9] of cases. Studies with more favourable outcomes report response rates of 63% [113], 67% [87] and 80% [62]. However, contrastingly, non-response rates of 81% [63] and 71% [112] were found by other publications. A recent systematic review of RCTs reported remission with RTX in 46% of SRNS (63% with MCD and 39% with FSGS) and sustained remission among responders in up to ~ 94% [58]. However, an open-label RCT in patients with refractory SRNS by Magnasco et al. demonstrated no additional benefit of RTX over CNI [63].

There is a recent study by Fujinaga et al. reporting 100% complete remission in children with SRNS receiving RTX [41]. This study is noteworthy because the authors emphasized the significance of timing of RTX initiation in SRNS patients. In the study, the authors investigated long-term outcomes after early application of RTX in six Japanese children who were unresponsive to a combination of CSA and intravenous methylprednisolone pulses [41]. The patients had RTX treatment within 6 months of disease onset (median 11 weeks), followed by retrial of high-dose intravenous methylprednisolone (2 mg/kg/d) and oral prednisolone and then switched to maintenance oral immunosuppressants (MMF or CSA). Using this protocol, all six patients achieved complete remission at a median of 158 days and maintained remission (although there were relapses) for a follow-up period of 5.1 years, without developing renal insufficiency [41]. In the previous open-label RCT in patients with refractory SRNS by Magnasco et al., two standard doses of RTX were not able to induce remission within 3 months after administration [63]. It is important to note that in the study by Fujinaga, most patients (4/6, 67%) achieved remission after 3 months of RTX administration with repeated doses (one patient required eight doses of RTX until complete remission was achieved) [41]. The authors suggested that in case of unresponsiveness, repeated administration over a longer period of time can be effective in SRNS. Evidence supports the concept that serum RTX levels may decrease more rapidly in SRNS due to persistent urinary losses [114, 115]. In that sense, it appears to require more doses in SRNS patients who have uncontrolled proteinuria [41, 116]. Moreover, the time from diagnosis of SRNS to the first RTX infusion was 2.5 years in the RCT by Magnasco et al. [63] and 2.2 years in the study by Topaloglu et al. [111], while it was 11 months in Fujinaga et al.’s study [41]. It may be speculated that a long-lasting nephrotic state leads to irreversible histologic changes, such as fibrosis and glomerulosclerosis [41], which results in imperfect response to RTX treatment. This view was supported by an observational study by Kamei et al., in which seven out of ten (70%) SRNS patients responded after repeated doses of RTX followed by intravenous methylprednisolone pulses and an additional oral immunosuppressant [62]. In this study, the seven patients who received RTX within 6 months of disease onset achieved complete remission, while two patients who had a longer duration of disease (61 and 121 months, respectively) progressed to end-stage kidney disease [41, 62].

Although rituximab is generally safe and well tolerated in most children, there are potentially serious adverse events that require caution: hypogammaglobulinemia, late-onset neutropenia, hepatitis B reactivation, serious infusion-related adverse events and infections, the latter with potentially fatal outcome with reports of *Pneumocystis jirovecii* pneumonia and progressive multifocal leukoencephalopathy [8, 58, 59, 61, 62, 117, 118]. In the study by Fujinaga et al., although all six patients achieved complete remission, four of the six had hypogammaglobulinemia requiring intravenous immunoglobulins (IVIG), and one of them developed persistent hypogammaglobulinemia demanding regular IVIG treatment (despite re-emergence of B-cells in the blood) [41]. Such hematologic adverse effects are reported more often and more severely in younger children. In previous studies with SRNS [41] as well as with SDNS [119], those patients who developed severe neutropenia and hypogammaglobulinemia were aged under 10 years. In addition, in a Japanese multicentre study involving 114 children, the median age was significantly younger in patients with more severe hematologic adverse effects (6.4 vs. 11.5 years) [120]. On the other hand, Bonanni et al. reported only 2 cases of severe neutropenia out of 100 Caucasian children with multidrug-resistant nephrotic syndrome who received RTX [121]. This discrepancy may be explained by the older age of patients in Bonanni’s group (median 9.2 years). Although the mechanisms leading to more complications in younger children are not well understood, younger age appears to be a particular risk factor for these adverse events.

To summarize, although RTX treatment in SRNS may not be as effective as in SSNS, early repeated administration with higher cumulative doses can be efficacious in the management SRNS. Early administration within 6 months of disease onset and a trial of multiple doses followed by methylprednisolone pulses with high-dose prednisolone showed favourable renal outcome. Adverse hematologic effects occur more frequently in children aged under 10.

### Ofatumumab

Ofatumumab, a novel humanized anti-CD20 monoclonal antibody, is an alternative to RTX in patients with anti-RTX antibodies or RTX hypersensitivity. It has also been used in patients with resistance to RTX [64, 67, 68] including cases with post-transplantation RTX-resistant SRNS [64, 122]. Ofatumumab successfully achieved remission in paediatric SRNS with severe adverse reactions to RTX [65] and anti-RTX antibodies [66]. Efficacy of ofatumumab so far is exceptionally positive; its first use reported complete or partial remission in five out of five children with SRNS [67] and in a more recent study in four out of four children with SRNS who completed ofatumumab treatment [64]. In the latter study, two patients experienced hypersensitivity to ofatumumab and received desensitization [64]. In a study with lower doses of ofatumumab, two out of four patients who were resistant to a combination or steroids, CNIs and MMF, achieved remission [68].

The side effects, however, were more prevalent with ofatumumab than RTX in the study by Bonanni et al. [121]. They investigated treatment outcomes in steroid- and multidrug-dependent nephrotic syndrome using RTX in 137 and ofatumumab in 37 patients. While treatment efficacy was comparable, infusion reactions, such as skin rash, fever, dyspnoea and late adverse effects, were more frequent in the ofatumumab treatment group [121]. In this study, immediate infusion reactions were effectively controlled by pretreatment including steroids, antihistamines and paracetamol, and salbutamol was critical for preventing respiratory complications.

In short, ofatumumab is an emerging substitute for RTX-resistant and RTX-hypersensitive patients with multidrug-resistant SRNS, although adverse effects and infusion reactions are more common than following RTX administration. Addition of a beta-mimetic agent in the pretreatment protocol was helpful in controlling respiratory reaction.

## Other monoclonal antibodies

There are a handful of novel monoclonal antibodies which have been used as potentially effective measures in nephrotic syndrome. Most of these, however, have been ineffective in SRNS, and only a few studies exist on paediatric patients.

### Abatacept/Belatacept

Abatacept, a cytotoxic T-lymphocyte-associated protein 4 (CTLA-4) immunoglobulin (Ig), blocks the CD28:CD80/CD86 co-stimulatory pathway and has recently been introduced as a treatment option for FSGS. A study by Yu et al. showed that B7–1 (CD80) overexpression was induced in podocytes in primary and recurrent FSGS and reported the efficacy of abatacept in four post-transplantation patients (two adults and two children) with recurrent FSGS refractory to immunosuppression including rituximab, and one adult patient with FSGS in the native kidney [69]. Controversies remain, since B7–1 (CD80) expression on podocytes has not been reproducible by different research groups [123, 124] and further research has showed no efficacy of abatacept in the management of FSGS/SRNS [70, 125]. Further prospective studies are on their way to prove or disprove the efficacy of abatacept in SRNS. The effects of belatacept, an agent with a higher potential to block B7–2 (CD86), also need to be further addressed.

### Adalimumab

Adalimumab is a monoclonal antibody blocking TNF-α [26]. In the FONT phase 1 trial of adalimumab, four out of ten patients (mean age, 16.8 ± 9.0 years) with primary FSGS showed a > 50% reduction in proteinuria, achieving partial remission during a 16-week treatment course [71]. To further test the hypothesis of adalimumab efficacy in FSGS, the FONT study group performed a phase II trial including 21 patients (14 children and 7 adults) with primary FSGS [72]. In the phase II trial, however, none of the subjects assigned to adalimumab achieved a 50% reduction in proteinuria [72]. These findings suggest that adalimumab may not be effective in the management of FSGS/SRNS.

### Fresolimumab

Fresolimumab is a monoclonal antibody targeting transforming growth factor-β (TGF-β). Sustained overproduction of TGF-β has been implicated in the pathogenesis of fibrosis, including human fibrotic kidney diseases and FSGS [73]. In a phase I clinical trial, complete or partial remission was achieved in 3 out of 16 adults (mean age, 37 ± 12 years) with primary FSGS who were previously unresponsive to CNIs or high-dose steroids, showing a response rate of 18% [53].

In the subsequent phase II trial with fresolimumab for 112 days, however, none of the 36 patients showed a > 50% decline in proteinuria compared to baseline [73]. The study was prematurely terminated due to futility, since a total recruitment of 88 patients was planned [73]. Three patients had some degree of decline in proteinuria but not meeting the criteria for remission, and fresolimumab was generally well tolerated [73]. Although the studies may have been underpowered, fresolimumab is no alternative in treating SRNS.

## Non-immunologic therapies

### Renin angiotensin aldosterone system (RAS) inhibition

Treatment with drugs that inhibit the renin-angiotensin axis has been recommended for children with SRNS by most of the previous reviews [7, 8, 26]. The response rate of RAS inhibition to lower proteinuria is reportedly around 40–50% in children with SRNS [9, 54, 56]. Furthermore, an RCT in 25 children with SRNS compared the efficacy of enalapril starting at low dose vs. starting at high dose [54]. The patients received concomitant steroids but no other immunosuppressants. In this study, the high-dose enalapril group showed significantly higher rates of proteinuria reduction (33% vs. 52%). Another RCT involving 45 children with SRNS demonstrated that fosinopril in combination with prednisolone significantly reduced proteinuria compared to prednisolone monotherapy [126]. In addition, there was a case report showing that RAS inhibition can be powerful in infantile SRNS as well, in which the authors reported complete remission by using RAS inhibition and supportive care in a 9-month-old patient with FSGS unresponsive to immunosuppression [127]. The patient was screened negative for *NPHS1*, *NPHS2* and *PLCE1* and was not tested for other genes at that time.

In short, angiotensin-converting enzyme inhibitors or angiotensin II receptor blockers are favourable for children with SRNS and are recommended by current guidelines [12, 31].

### Sparsentan

Endothelin type A (ETA) receptor antagonists have emerged as promising therapies that may enhance RAS inhibitory action [79, 128]. Sparsentan is a dual endothelin type A (ETA) and angiotensin II type 1 receptor antagonist.

A randomized, double-blind, active-control, dose-escalation study (DUET), a phase II study, compared the efficacy and safety of sparsentan against irbesartan in 96 patients (aged 8–75 years) with primary FSGS [79]. The results showed that sparsentan-treated patients had greater reductions in proteinuria than the irbesartan-treated group after 8 weeks of treatment [79]. Adverse events of sparsentan were comparable to irbesartan, showing that sparsentan was generally safe and well tolerated [79]. Although it requires further clinical experience, this novel agent seems to be a promising non-immune modulating option for SRNS. Data focusing on paediatric patients are needed in the future.

### Galactose

Galactose might reduce proteinuria by binding to putative circulating factors and inhibiting their activity [26, 78, 129]. Case reports in children and adults suggest that patients with FSGS/SRNS may benefit from orally given galactose [74, 75, 129]. A prospective clinical trial using galactose in seven children with SRNS demonstrated histological improvement in kidney biopsies but no effect in reducing proteinuria [76]. Nevertheless, a more recent phase II clinical trial showed that three out of seven FSGS patients treated with oral galactose showed at least a 50% reduction in proteinuria at 6 months after initiation and a sustained effect for 3–12 months after discontinuation of galactose [72], which raised the possibility of galactose as an adjuvant agent in treating SRNS (Table 1).

### ACTH analogues

The exact mechanism of adrenocorticotropic hormone (ACTH) in reducing proteinuria is not well elucidated. In an observational study, application of an ACTH analogue in gel form reduced proteinuria in 11 out of 21 patients with nephrotic syndrome [130]. However, it should be noted that subjects had a variety of different immunologic kidney diseases, including membranous nephropathy, membranoproliferative nephropathy minimal change disease, immunoglobulin A nephropathy and lupus nephritis [130]. Intramuscular application of low-dose ACTH has also been reported to be effective in a study of 18 adults with nephrotic syndrome, inducing complete remission in 33% (6/18) and partial remission in 22% (4/18) [131]. In contrast, a recent paediatric RCT – The ATLANTIS randomized trial for adrenocorticotropic hormone for childhood nephrotic syndrome – has reported that twice-weekly administration of ACTH (80 U/1.73m^2^) was not effective in preventing relapses in children with FRNS or SDNS [132]. With regard to SRNS, one study with 24 FSGS patients reported complete or partial remission in 29% (7/24) after initiation of ACTH gel treatment, suggesting its possibility as a treatment option [77]. However, the high expense of ACTH, the relative lack of evidence [133] and recent reports on its prescription having been influenced by financial conflict of interest [134, 135] should prevent hasty recommendations on this drug being made.

### Mesenchymal stem cells (MSCs)

Mesenchymal stem cells (MSCs), known for their immunomodulatory and anti-inflammatory effects, have been considered as a potential therapeutic agent for treating immune-related diseases, including nephrotic syndrome [136, 137]. Currently, a phase I open-label pilot study on safety and efficacy is ongoing (Allogenic AD-MSC Transplantation in Idiopathic Nephrotic Syndrome (NCT02382874)).

### Retinoids

Retinoids are analogues of vitamin A that regulate cellular differentiation. It has been suggested that retinoids are capable of restoring podocyte structure in that they reduce proteinuria in animal models of kidney diseases [138]. A recently completed phase II trial on the efficacy of retinoids in patients with FSGS, however, reported no reduction in proteinuria in seven patients who completed isotretinoin treatment (NCT00098020).

## Treating monogenic SRNS

Although monogenic SRNS is thought to be inherently non-immune-based, some cases have been reported to at least partially respond to immunosuppression [27, 139–144]. Moreover, there are familial cases reported as monogenic SSNS or mixtures of SSNS and SRNS [15, 145–147]. A recent review by Saleem has addressed this issue and proposed some mechanisms that explain therapeutic efficacy in monogenic nephrotic syndrome: (1) the affected genes may play a role in the immune responses, or (2) mutations in podocytes may alter their response to immunosuppressants [15]. In addition, direct effects of corticosteroids [148] and CSA [149] on podocytes may explain the variable responses to immunosuppression in monogenic nephrotic syndrome.

Regarding the efficacy of CSA in genetic SRNS, there have been further reports from a few cohorts. Buscher et al. studied immunosuppression outcomes in 91 children with SRNS (including congenital nephrotic syndrome) and reported that patients with Wilms tumour suppressor gene 1 (*WT1*) mutations were more responsive to CSA [150]. They further compared the response to CSA in non-genetic and genetic SRNS in a larger scale involving 231 patients (131 monogenic SRNS including 60 congenital nephrotic syndromes) [151]. Therein, they documented response to CSA in 19% of monogenic SRNS (3% complete remission and 16% partial remission). In the PodoNet registry report, there were 74 patients with documented genetic SRNS, and 14 patients (19%) showed response to immunosuppression [9].

Specific gene disorders can sometimes benefit from new therapeutic strategies. Mutations in the genes related to biosynthesis of Coenzyme Q 10 (CoQ_10_, ubiquinone) cause primary CoQ_10_ deficiency resulting in various clinical phenotypes. A portion of these patients present with SRNS, often with extrarenal manifestations such as sensorineural hearing loss or neurologic deficit [152–156]. In monogenic SRNS related to primary CoQ_10_ deficiency, early initiation of CoQ_10_ supplementation has been reported to reduce proteinuria and delay disease progression [157, 158].

## Conclusions

SRNS is a heterogeneous entity, and the treatment effects may differ by the aetiology. For immune-mediated SRNS, based on the results from prospective studies and RCTs, the combination of a CNI and an alternate-day corticosteroid may be the major strategy. TAC appears to achieve a higher remission rate compared to CSA and a lower degree of side effects including nephrotoxicity and gingival hypertrophy. MMF seems not superior to CNI as a monotherapy, but its use in combination with other measures may be considered in patients with CNI-resistant or CNI-dependent SRNS. RTX is reported to be less effective in SRNS, but timely administration with repeated dosing has shown to induce favourable outcomes. For monogenic SRNS, CNIs can be used although not as efficacious as in immune-based SRNS. Monogenic SRNS associated with CoQ_10_ deficiency may benefit from CoQ_10_ supplementation. Other novel agents require further validation for efficacy and safety in managing children with SRNS.
